# The Correlation of Self-efficacy and Social Support with Social Participation: A Cross Sectional Study among the Elderly

**DOI:** 10.25122/jml-2019-0010

**Published:** 2019

**Authors:** Nasrin Hosseingholizadeh, Roya Sadeghi, Hassan Eftkhar Ardebili, Abbas Rahimi Foroushani, Mohammad Hossein Taghdisi

**Affiliations:** 1. Department of Health Education and Promotion, School of Public Health, Tehran University of Medical Sciences, Tehran, Iran; 2. Department of Epidemiology and Biostatistics, School of Public Health, Tehran University of Medical Sciences, Tehran, Iran; 3. Department of Health Education and Promotion, Iran University of Medical Sciences, Tehran, Iran

**Keywords:** Social participation, elderly, self-efficacy, social support

## Abstract

There is ample evidence that a key contributor to healthy aging is social participation; thus, identifying its determinants can help to improve it. The current study aimed at assessing the relationship between social support and general self-efficacy with social participation. This cross-sectional study was conducted on 456 (male: 237, female: 219) older adults, aged 60-74 years, who were the member of community centers in Tehran, Iran, in 2016. The multi-stage cluster sampling method was employed to select the participants. Primarily, Tehran was divided into five areas (i e, North, South, East, West, and Center). Then, two community centers were randomly selected from each area. Participants with the ability to perform their daily activities independently were enrolled in the study. Participants responded to four self-report questionnaires: socio-demographic, social participation, social support, and general self-efficacy. The majority of the participants were within the age range of 62–68 years with the maximum proportion of social participation (mean ± standard deviation (SD): 37.69 ± 8.34). Findings of the current study indicated that gender, education level, reading books, newspapers, or magazines (p <0.001), living area, living arrangement, and occupational status (p<0.05) were associated with social participation. Multiple linear regression adjusted for living area, sex, and educational level showed that self-efficacy (β= .08; p<.001) and social support (β= .64; p<.001) predicted social participation. Findings showed the importance of social support and self-efficacy in social participation. Also enhancing the literacy of seniors should be given priority.

## Introduction

The elderly population is growing rapidly worldwide. According to the World Health Organization (WHO) report in 2015, a total of 900 million people are 60 years and above in age [1]. Elderly population in Iran in 2016 was 5% but it is going to grow to 20% in 2050 [2]. Consequently, geriatric diseases that hinder the elderly from active engagement in the society also increase. Social participation ranging from preparatory home activities to society assistance [3], is a good option to track this problem. Evidence regarding the beneficial effects of social participation on health is reported in many studies [4–11]. Variables studied in the current study also have a relationship with health. Social support is associated with health[12], health promoting behaviors [13], and functional capability [14]. It was also acts as a mediator between social participation and health, based on a review study [15]. Self- efficacy, the other variable, was greatly related with vigor and memory [16].

The first reason for addressing the issue of social participation in Iran is related to low level of social participation among the elderly [17]. Another one is concerned with income, literacy and life habits. Factors which greatly influence social participation [18], according to a recent research in Iran more than half of older adults were illiterate, didn’t get allowance. Furthermore, around 40% of them spend their free time at home [19]. The last, has to do with reduction in the network size in old age [20], a phenomenon which is linked with social participation [21]. Previous studies confirmed association between social participation and quality of life [22, 23], social capital [24, 25], environmental factors [26, 27], and social support [28–30].

This study was to examine the effect of self- efficacy and social support together on social participation. So far, no study had examined the joint effect of the above mentioned variables. Similar studies had examined the effect of these variables on physical activity. For instance, Anderson et. Al., [2006] showed that they could predict physical activity [31]. Another study among 12–16 year old students found that self- efficacy for physical activity and friend support predicted physical activity intention. It is worth noting that the effect of self- efficacy was more powerful [32]. A study by Warner et.al[2011] confirmed that exercising self-efficacy and social support in the elderly predicted physical activity; in addition, the two variables interacted with each other [33]. The current study showed that self-efficacy predicted social participation. Similarly, other studies in Spain and the US by Perkins et.al [2008] revealed that exercising self-efficacy predicted physical and social activities [34]. A study by Toepoel [2013] showed the role of different persons such as family and friends in stimulating social activities [35].

## Materials and Methods

### Sample selection

The current cross-sectional study was conducted on 456 older adults aged 60-74 years, who were members of community centers in Tehran, Iran, in 2015. The participants were selected using the multi-stage cluster sampling method. At first, Tehran was divided into five geographical zones, and then two community centers were randomly selected from each zone. Afterwards, the elderly with the ability to perform their daily activities independently were selected to participate in the study. A verbal consent was obtained from all participants. Participants that did not attend the neighborhood centers were substituted.

At the beginning, the participants were informed about the study objectives and ensured about the confidentiality of their information. Questionnaires were completed via interviews by two trained research assistants. The sample size is computed as follows:

**Figure:**
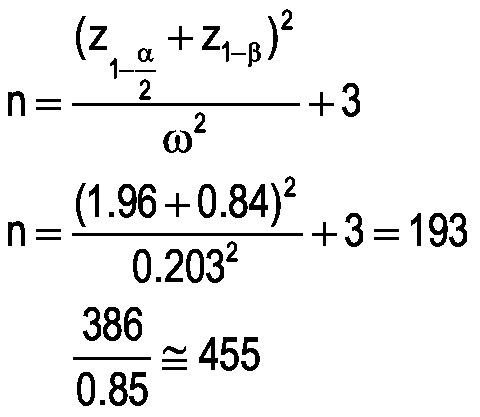


The sample size formula for the correlation coefficient was used so that with 95% confidence interval (CI), and 80% test power, if the correlation coefficient is 0.2 or higher, a statistically significant difference is obtained. Due to interdependent answers, the sample size was multiplied by 2; attrition rate was considered 0.15.

### Research instrument

The selection of instruments used to collect data in this study:

#### Socio- demographic form

The variables evaluated in this form were: age group (60-61, 62-68, >69 years), gender, marital status (married, etc.), educational attainment (reading and writing ability, primary school, junior high school, diploma, bachelor or higher degree, employment status (employed, unemployed), living arrangement (spouse, spouse and children, etc.), living area (north, south, east, west, and center of Tehran), watching TV, listening to radio (yes/no), reading books (yes/no), newspapers, or magazines (yes/no).

#### Social participation questionnaire

The questionnaire consisted of 22 items. The items were extracted by reviewing the relevant literature [36–40]. The frequency of attending social activities was asked from the participants. Responses were never, at least once a year, at least once a month, at least once a week, and at least once a day, which were scored based on a five-point Likert scale from 0-4. The total score was calculated by summing up the items scores (max = 88). The content validity of the questionnaire was tested in a pilot test on 30 elderly members of a community center. They were also asked to read and comment on the questions. To obtain content validity index (CVI) and content validity ratio (CVR), the questionnaire was delivered to two expert groups separately. Confirmatory factor analysis (CFA) with AMOS version 22 was also performed to assess structural validity. The psychometric properties of the social participation questionnaire were as follows: the mean scores of CVR and CVI were 0.8 and 0.9, respectively. CFA for the social participation questionnaire showed acceptable values (*χ2/df* < 3, GFI (Goodness of fit index), NFI (Normed-Fit Index) >0.9, and *RMSEA* (Root Mean Square Error of Approximation <0.08. Its reliability was measured by intra-class correlation (ICC) ranging 0.72-0.95.

#### Social support questionnaire (the Sarason short-form)

The social support questionnaire was first developed by Sarason et al. The short-form was also validated by him in 1987 [41]. The questionnaire consists of six 2-part questions. The first part measures social support by asking the number of people that offer them help (maximum 9) and the second, asks the satisfaction with the received support (maximum 6). To calculate the total score, the mean values of each part are calculated. As the two parts were strongly correlated in the analysis, to eliminate the collinearity effect, the second part was omitted from the analysis. Psychometric properties of the complete version of social support questionnaire were confirmed previously in a study in Iran [42]. Thus, for validity confirmation, construct validity was used. Exploratory factor analysis (EFA) for the questionnaire showed one factor with 78% prediction of variance. Cronbach’s alpha reliability of the questionnaire was 0.94.

#### General self-efficacy scale

Self-efficacy is defined as believing in self-competence to deal with new tasks and adapt to difficulties when encountering stressful and challenging events. It is associated with self-esteem, happiness, success, and satisfying social relationships [43]. General self-efficacy scale was developed by Schwarzer and Jerusalem, and a study in Iran proved its good psychometric properties [44]. The general self-efficacy scale consists of 10 questions, which scored based on a four-point likert scale ranging from 1(not correct)-4 (completely correct).

#### Data analysis

To analyze the data, *t* test, analysis of variance (ANOVA), exploratory factor analysis (EFA) and CFA, multiple linear regressions were conducted with SPSS version 22 and AMOS version 22 software programs. p-value of <0.05 was considered significant.

## Results

The participants consisted of 52% males (n=237) and 48% females (n=219). The results showed that most of the respondents were in the age range of 62-68 years (47.4%) with high level of social participation (mean: 37.69 ± 8.34). The majority of the participants was married (57.2%), had low educational attainment (56.1%), and lived with spouse and children (43.2%). Most of the respondents were unemployed and described their walking path around their home as easy (78.5%); watching TV was more popular than listening to radio (93% vs. 43%); nearly 70% of the respondents did not read books, newspapers, or magazines (Table 1).

**Table 1:** Relationship between socio- demographic characteristics and social participation

Variable	N (%)	Mean±(SD) of social participation	F or t-test	p_value
**Age group**			1.305	0.272
**60–61**	122(26.8)	36.37(7.5)		
**62–68**	216(47.4)	37.69(8.34)		
**69<**	109(23.9)	36.5(8.82)		
**Sex**			3.522	< 0.001
**Male**	237(52)	38.48(7.87)		
**Female**	219(48)	35.79(8.45)		
**Marital status**			–1.892	0.059
**married**	343(57.2)	35.92(7.98)		
**Not married**	113(24.8)	37.61(8.32)		
**Educational attainment**			5.102	< 0.001
**Reading and writing**	141(30.9)			
**Primary school**	115(25.2)	36.94(8.19)		
**Junior high school**	60(13.2)	37.91(7.98)		
**Diploma**	87(19.1)	37.85(7)		
**Bachelor/higher degree**	52(11.4)	41.06(8.7)		
**Employment status**			–2.354	0.190
**Employed**	98(21.5)	38.92(7.54)		
**Unemployed**	358(78.5)	36.71(8.39)		
**Living arrangement**			3.26	0.039
**Spouse**	136(29.8)	38.09(7.9)		
**Spouse and children**	197(43.2)	37.2(8.69)		
**Else**	122(26.8)	35.67(8.19)		
**Living area**			4.098	0.003
**North**	76(16.7)	38.3(9.14)		
**South**	117(25.7)	37.62(8.06)		
**East**	57(12.5)	33.77(7.52)		
**West**	109(23.9)	36.41(8.02)		
**Center**	97(21.3)	38.89(7.93)		
**Watching TV (Regularly)**			–1.952	0.052
**Yes**	426(93.4)	37.39(8.19)		
**No**	30(6.6)	34.35(8.86)		
**Listening to radio**			–1.296	0.195
**Yes**	195(42.8)	37.77(8.50)		
**No**	261(57.2)	36.75(8.06)		
**Reading book (regularly)**			–5.737	
**Yes**	141(30.9)	40.4(8.60)		
**No**	315(69.1)	35.75(7.69)		
**Reading news paper or magazine (regularly)**			–4.88	< 0.001
**Yes**	139(30.5)	39.97(7.67)		
**No**	317(69.5)	35.97(8.22)		

There was a significant relationship between social participation and gender (t = 3.522, p <0.001), educational attainment (F = 5.102, p <0.001), reading books (t = -5.737, p <0.001) newspapers or magazines (F = -4.88, p <0.001), living area (F = 4.098, p = 0.003), living arrangement (F = 3.26, p = 0.039), and employment status (t = -2.354, p = 0.019); however, age, marital status, walking path, watching TV, and listening to radio were not significantly related to social participation (Table 1).

The most frequent activity was helping others, while the least was going to a library or cultural center. Taking care of children was reported in 60% of the elderly. Around 40% of the participants did outdoor sports, and 57.5% of the participants travelled at least once a year (Table 2).

**Table 2:** Frequency of social participation activities

Items	Every day n(%)	At least once a week n (%)	At least once a month n (%)	At least once a year n (%)	Never
**Visiting family (or visited by them)**	204(44.7)	183(40.1)	43(9.4)	21(4.6)	5(1.1)
**Visiting friends or relatives (or visited by them)**	88(19.3)	147(32.2)	154(33.8)	57(12.5)	10(2.2)
**Calling to family**	296(64.9)	108(23.7)	26(5.7)	3(.7)	22(4.8)
**Calling to friends, relatives or neighbors**	162(35.5)	151(33.1)	104(22.8)	7(1.5)	31(6.8)
**Calling to friends, relatives or neighbors**	162(35.5)	151(33.1)	104(22.8)	7(1.5)	31(6.8)
**Walking outside for 20 minutes or more**	340(74.6)	55(12.1)	18(3.9)	4(.9)	39(8.6)
**Shopping grocery, drug or other home requirements**	233(51.1)	136(29.8)	36(7.9)	7(1.5)	44 (9.6)
**Going to cinema, theater and concerts**	0	2(0.4)	26(5.7)	54(11.8)	374(82)
**Going to museums and exhibitions**	0	4(0.9)	21(4.6)	68(14.9)	363(79.6)
**Going to restaurants and coffee shops**	6(1.3)	12(2.6)	89(19.5)	102(22.4)	247(54.2)
**Participating in outdoor sports**	57(12.5)	54(11.8)	37(8.1)	37(8.1)	271(59.4)
**Travelling along with family or by tours**	1(.2)	8(1.8)	70(15.4)	262(57.5)	115(25.2)
**Participating in training courses**	0	9(2)	10(2.2)	18(3.9)	419(91.9)
**Writing (books, letter, story or an article for newspaper)**	2(0.4)	9(0.2)	8(1.8)	11(2.4)	426(93.4)
**Doing artistic activities (singing, drawing….)**	7(1.5)	16(3.5)	13(2.9)	14(3.1)	406(89)
**Taking hobby (gardening, hunting or fishing)**	12(2.6)	9(2)	15(3.3)	23(5)	397(87.1)
**Cooperation with neighbors**	4(0.9)	74(16.2)	137(30)	74(16.2)	167(36.6)
**Helping others if needed**	12(2.6)	158(34.6)	191(41.9)	48(10.5)	47(10.3)
**Presence in mosque, shrines or holy places**	166(36.4)	108(23.7)	72(15.8)	31(6.8)	79(17.3)
**Participating in congregations and ceremonies**	0	8(1.8)	149(32.7)	258(56.6)	41(9)
**Activity in associations (politic, religious and so on)**	14(3.1)	48(10.5)	43(9.4)	45(9.9)	306(67.1)
**Going to library or cultural centers**	9(2)	19(4.2)	13(2.9)	13(2.9)	402(88.2)
**Taking care of children or another person**	89(19.5)	108(23.7)	56(12.3)	20(4.4)	183(40.1)

Table 3 presented the predictive effect of self-efficacy and social support on social participation. For this purpose multiple linear regression, with backward stepwise regression approach, was used. Other confounding variables which remained in the final model were living area, education level, and sex. According to the results, self- efficacy (β = 0.08; SE: 0.02; p<.001) and social support (β = 0.64; SE: 0.16; p < .001) predicted social participation. Also social participation level in all areas, except west, was different with east area. Males had higher social participation than females (β = –2.15; p < .003). Regarding the education level, only people with bachelor’s degree and higher had different social participation (β = 4.78; p < .001).

**Table 3:** Multiple linear regression model for social participation

Variables	n (%)	Mean of SP (SD)	B (SE)	p-value+
**Living area**				
East(reference)	57(0.037)	33.77(7.52)	—	
North	76(0.166)	38.04(9.14)	3.16(1.36)	0.02
South	117(0.25)	37.63(8.04)	4.55(1.27)	<0.001
West	109(0.23)	36.41(8.02)	1.66(1.27)	0.190
Center	97(0.212)	38.89(7.94)	4.57(1.29)	<0.001
**Sex**				
Male(reference)	237(0.519	38.49(7.88)	—	
Female	219(0.48)	35.79(8.46)	–2.15(0.73)	0.003
**Education**				
Low literacy &primary (reference)	256(0.56)	36.06(8.38)	—	
Guidance	60(0.13)	37.92(7.98)	1.36(1.11)	0.222
Diploma	87(0.19)	37.85(7.00)	1.91(1.00)	0.057
Bs and higher	52(0.11)	41.06(8.71)	4.78(1.22)	<0.001
**Self- efficacy**	—	—	0.08(0.02)	<0.001
**Social support**	—	—	0.64(0.16)	<0.001
**Total**	456(100)	37.22±8.26	—	—

## Discussion

This study confirmed that social support and self- efficacy predicted social participation though the effect of social support was stronger. Regarding socio-demographic characteristics, findings of the current study showed a significant relationship between social participation and gender, educational level, employment, household arrangement, and living area. Another study conducted by Asadollahi (2011) in Iran confirmed the current study findings [45]. Also, a study in Canada conducted by Richard [2009] et. al., corroborated the current study findings concerning the association between gender, educational level, household arrangement, and social participation. In line with the current study results, marital status was not related to social participation in that study [46]. A study in Iran, by Darvishpoor et.al., [2014], which was conducted among above 60-year-old elderly, confirmed our study findings about low level of social participation and more social participation among males, and well-educated people [17]. It was noted that social participation was significantly different in various areas, which might be caused by various patterns of social participation in different places or socioeconomic differences. A study in Japan conducted by Sewo Sampaio [2013], for instance, showed that people in rural areas often did physical work activities, whereas people from urban areas more often were busy with reading, writing, and contacting their friends [47]. Contrary to our study finding, Levasseur et.al., [2015] didn’t find social participation difference between distinct areas [26]. According to findings of the current study, social participation was higher in employed individuals and men. It can be justified by considering the routine of males and females` lives. Males are usually employed and attend social activities more since they should earn money for the family and this condition continues until the old age. A study by Hyyppä et. Al., [2008] indicated that social participation is steady overtime [48]. Watching TV and listening to radio were not significantly related to social participation in the current study, which was in line with the findings of a study by Toepoel [2013]. [35] Findings of the current study showed that the most frequent activities among the elderly were helping others, visiting friends or relatives, and participating in congregations and ceremonies. Likewise, another study in Tehran by Fathi [2011], demonstrated that informal cooperative participation was more prevalent than formal participation [49]. The reason for the dominance of informal participation among the elderly is the tendency of old people to be sedentary; therefore, their social interactions take place indoors instead of outdoors. Outdoor sports should be encouraged more in the elderly since most of them never participated in outdoor sports. Approximately half of the participants did not go to restaurants and cafes, and 57.5% of the participants travelled at least once a year, which reflected income status or lifestyle of participants in the current study. Religious participation is an important factor in social cohesion and most participants in the current study stated that they visited holy places, but their engagement in associations was poor [50]. In sum, the elderly attended informal social activities more than formal ones. Since most of older adults in the current study were illiterate, activities such as training courses, writing, and going to a library were rare among them. Also, artistic activities and hobbies were not common.

## Conclusion

Previously no study has examined the relationship between general self- efficacy and social support with social participation. Since causative relationships cannot be inferred from cross sectional studies, findings of the current study should be interpreted cautiously. Second, due to the self-report nature of the results, answers might have some bias. To determine the exact effects of the studied variables on social participation, longitudinal studies should be conducted. Also, studying other related factors such as income or personality can be useful. As most participants in the current study were illiterate, and educational level and social participation were related, improving literacy in elderly population is important. This study found that social participation was low among the elderly, so policy makers should pay more attention to it. Identifying other social participation determinants needs more research. One important point found in relevant literature is lack of qualitative studies in this field.

## Acknowledgements

The current study protocol was approved by the Ethics Committee of Tehran University of Medical Sciences, Tehran, Iran (No. 1-9021108005). The authors appreciate Tehran University of Medical Sciences for funding the project and the Socio-Cultural Deputy of Tehran Municipality that permitted attending neighborhood centers. Also, the authors thank the research assistants as well as the elderly who participated in the study.

## Conflict of Interest

The authors confirm that there are no conflicts of interest.
